# Inoculation in Yellow Fever

**Published:** 1855-10

**Authors:** M. Morton Dowler


*Humboldt's Viperine Inoculation for the prevention of Yellow Fever:* Translated by M. Morton Dowler, M. D., from the *Gaz. Heb. de Med.*, of June 29, 1855, with remarks.

"Several American and Spanish journals bring us new details relative to *inoculation with the venom of the viper as a preventive means against the yellow fever*, with respect to which we have already taken occasion to offer some remarks. The new world has gone mad with the discovery. The discoverer, Dr. Humboldt, nephew of the celebrated *savant* of this name, is followed wherever he goes with praises and benedictions; and the Captain General of Cuba has authorized him to open a special establishment for inoculation, by a decree which trenches very remarkably by its caution on the confident enthusiasm of the public. The following is the wording of the decree:

'I have received the application which has been made to me by Dr. William Humboldt, asking an authorization to create a public establishment in order to experiment with the inoculation of the preventive virus of Yellow fever, in which persons who wish to be inoculated will be taken care of till they are cured. I believe the time has not yet arrived in which we may be able to decide whether this virus does or does not truly protect against Yellow fever; but as the numerous inoculations made in the Military Hospital of this city has not produced any dangerous results, we see no reason for preventing Dr.

Humboldt from practising them under the conditions and form that he has prescribed. I, therefore, authorize him to originate the establishment that he desires to place at the disposal of the public. This establishment will be held to the general rules of inspection and medical police, to which the general infirmaries are subjected. CONCHA.

'HAVANA, February 9, 1855.'

In the memoir that he read before the Academy of Medical Sciences of Havana, M. de Humboldt himself gives the history of his discovery. He remarked, about seven years ago, that a number of criminals, who had been brought from the interior of Mexico to the bagnois of Vera Cruz and of St. Juan d'Ulloa, arrived there affected with the primary symptoms of yellow fever, and that in these cases the disease put on a character of special severity. Wishing to find out the conditions which thus gave birth to the disease before having any communication with the infected place, where it is ordinarily taken by the non-acclimated, he concluded to accompany from the interior to the lands of Vera Cruz the train of condemned criminals. Very soon he remarked that the yellow fever developed itself constantly as a consequence of the bite of a little viper which is very common in this country, which had ready access to the convicts, as they walked barefoot. Having collected a great number of these reptiles, he caused them to bite numerous dogs, all of which, at the end of three or four hours, exhibited the ordinary prodromena of yellow fever, and sunk with fœtid hemorrhages and signs of cerebral congestion. One could scarcely think of the employment of a prophylactic virus which produced such effects. It was necessary at least to mitigate it. With this object there occurred to M. de Humboldt the very singular idea, we must say, of causing the vipers to bite a piece of animal tissue, and allowing it to putrefy, to inoculate with the sanies. He selected sheep's liver. (This article of sheep's liver has been most happy; it is the substance which he chose to cure hemeralopia, as given in one of our preceding articles.) A piece of this hepatic tissue, weighing thirty grammes, was bitten by six vipers and allowed to putrefy, and then the liquor was inoculated into six dogs. Experience showed that by taking care not to insert the matter in more than from three to six punctures, nothing resulted from it but a slight febrile movement. The punctures neither inflamed nor suppurated. This is the practice which the author adopted on the human species.

The sanies above mentioned, was at first inserted under the skin of two criminals, four punctures to each. Cephalalgia, pains in the back, and fever, developed themselves almost immediately, and continued from four to twelve hours only, but the fever reproduced itself under the form of accession during the three or four days following. The innocuous character thus proved on man, induced him to continue his experiments. Of one hundred inoculated, belonging to the category of convicts or of persons sent to Europe, not one had the yellow fever; and, says the experimenter, statistics show that only four in one hundred of non-acclimated individuals who arrived at Vera Cruz, and there spend the summer, escape an attack of the scourge. Later in 1850, '51, '52, new experiments are made, the latest document showing the total number of inoculations to be 1,428. In seven cases only of these has the disease appeared in spite of inoculation, and these seven all recovered.

We may not forget to add, that in order to secure the inoculated from the alarming symptoms which are developed in some exceptional case, the author causes them all to use a mixture composed of the syrup of guaco, syrup of rhubarb, of gum-gutta, and iodide of potassium. The guaco (family of the corymbifera,) is celebrated in the cure of the bites of serpents, whence it follows that M. de Humboldt hastens, with all speed, to cure the patients that he has punctured; and for this we are far from blaming him.

Our object is merely to lay before the reader the progress of an event which has made more noise than did, in its origin, the discovery of Jenner. The reader will easily see for himself all that is wanting to the documents produced by M. de Humboldt in order to legitimize his pretensions. We wish with all our heart that these pretensions may be verified."

REMARKS.

The foregoing we translate from the "Gazette Hebdomadaire de Medecine et de Chrugie," of the 29th of June. Though the able editor, M. Dechambre, has discussed the subject in a rather semi-serious tone, exhibiting a well marked vein of doubt and incredulity, he has evidently intended to throw considerable interest around the subject of the Humboldtian inoculation. We may remark, *en passant*, that whatever future laurels may crown the brow or the yellow fever inoculator, they can confer no additional honors on the house of the illustrious Alexander Von Humboldt, as he himself declares that he has no nephews that bear his name. If, however, the inoculating Humboldt be worthy of belief; if his pretensions shall stand the uncompromising test of observation and experiment, then, indeed, the venerable savant might well be proud to adopt him, with a waiver of all questions of "propinquity and property of blood," as the greatest benefactor of the human race either ancient or modern—as the greatest of all men bearing the name of Humboldt.

We may, however, remark that the Baron Von Humboldt has been more annoyed than flattered by claimants to relationship sojourning in the western world, which has led him to request his friends of the press in New-York, in order to get rid of the annoyance, to publish to all the world that he has no relations afloat bearing his family name. While the genuine disciples of the venerated Baron are but unfortunately too few, those of another traveler and very different Baron with a German name and a lively imagination, are by no means wanting. We allude to Munchausen. The editor of the "Gazette" has immeasurably overrated the public enthusiasm in relation to the inoculator's pretended discovery—the alleged excitement being quite imaginary. The "praises and benedictions" have not yet become manifest. The inoculator has one highly important thing to do, and that is to show himself worthy of belief; and the excitement, "praise and benedictions" will forthwith appear. The tenor of the authorization granted by General Concha, leads us inevitably to discredit the inoculator's whole history, and to infer the entire falsity of his asserted Mexican experience. If the fourteen hundred and twenty-eight inoculations at Vera Cruz be not a sheer romance, why did not the inoculator on making application to his excellency, General Concha, lay before him authentic and positive proofs of those, amongst the most marvellous results ever related by man? It is plain that the inoculator obtained a mere temporary toleration, and that General Concha had personally no faith in the pretensions put forth, excepting his belief that the inoculation would not kill or prove dangerous. Surely his excellency must be *cautus homo et difficilis*, if the fourteen hundred and twenty-eight results in Mexico, well authenticated and sworn to by the authorities at Vera Cruz, and laid before him, could not carry some kind of conviction. The whole account of the criminals, the snakes, and the fourteen hundred and twenty-eight results, stand out at present as a veritable Munchausenism. The inoculator, during last summer, made his appearance in New Orleans, and in a long charlatanical manifesto, published in the city papers, proclaimed his discovery, and called on the unacclimated in this city to come and be "vaccinated," asserting in his advertisement that he had during seven years inoculated "more than two thousand persons," all of whom had been protected against the disease, rendered perfectly safe by the aid of his "anti-septic syrup." His later account, however, as given to the Medical Society at Havana, and taken as above, makes the number only fourteen hundred and twenty-eight, *six hundred less!* If the inoculator is no better at observing than at counting what becomes of our faith? We believe that he never inoculated any person in New Orleans, and we are very credibly informed that when closely pressed, he acknowledged that he had no such thing as a prophylactic, and that his advertisement was put forth merely to attract public attention! In fact we have only to read his testimony before the Sanitary Commission of New Orleans, to see that he pronounces his own advertisement a falsehood by maintaining the doctrine that, "Yellow Fever has no specific character, or pathognomonic symptoms, which can be defined in its cause, in its duration, or in its attributes; but that it is an accidental variety of a numerous and variable class of continuous and remittent fevers of certain latitudes; fevers from which it differs only by its violence, and the rapidity of its course, and its final phenomena. That its apparent causes, the principal and essential symptoms, and its pathological conditions, as also its periods of inception, of its greatest development, and of its decline, all proclaim that it is, like these other fevers, a variety of the same kind, that cannot with exactitude, or with the view of reconciling the facts in contradiction in the history of the tropics, be subdivided for any essential or appreciable difference in their multiform varieties."

The above is an extract from a long communication which he laid before the Sanitary Commission, proclaiming himself a nephew of the Baron Von Humboldt, and everywhere distinctly and clearly denying the specific character of yellow fever, but never once alluding to the snakes, the convicts, the sheep's liver or the inoculations, the whole communication necessarily by implication going to show that if snake's poison be good for yellow fever, it is equally good for any other fever, whether remittent, intermittent, or continued; the yellow fever being, according to him, no more a specific pestilence than the fever and ague. The Sanitary Commission* were sadly remiss in their newspaper reading, or on wiping their spectacles and taking up the newspapers laying on their table in the Municipal Hall, they would have found a long advertisement

*It may not be amiss to remark that, according to the established "Code of Medical Ethics," "no one can be considered as a regular practitioner, or a fit associate in consultation, whose practice is based on an exclusive dogma."

Again, chapter II.; sec. 4. "Equally derogatory to professional character is it for a physician to hold a patent for any surgical instrument or medicine; or to dispense a secret *nostrum*, whether it be the composition or exclusive property of himself or of others; for, if such nostrum be of real efficacy, any concealment regarding it is inconsistent with beneficence and professional liberality; and, if mystery alone give it value and importance, such craft implies either disgraceful ignorance or fraudulent avarice. It is also reprehensible for physicians to give certificates attesting the efficacy of patent or secret medicines, or in any way to promote the use of them."

in the public papers bearing the hand and seal of their witness, warning the people that the disease is altogether the reverse of what he had proclaimed it before the commission; that the disease is as specific in its chaaacter as the small-pox itself, and equally as amenable to prophylaxis. Why was this document withheld from the public? this testimony written and subscribed to? this solemn appeal to the people of New Orleans! "Silence is great," but not always! He appears in New Orleans playing two different roles with great versatility: the one as a theoretical assistant on behalf of the famous Sanitary Report; and the other as an advertising doctor, preaching a diametrically opposite doctrine as a mode of communing with the people. Either the inoculator's professional card, or his long communication laid before, and published by the Sanitary Commission, was coolly untrue; or else both were in the same category. No one reading both, can have any faith in the witness, or believe a word in relation to the snakes, convicts, dogs, sheep's liver, or inoculation. Besides, direct testimony from Havana goes to show that his inoculations have not the least prophylactic power whatever.

We hope our learned confrere of the "Gazette Hebdomadaire" will do everything in his power to calm and allay "les entrainements de la confiance publique," should they become as extensively epidemic in Paris as he has represented them in the new world.

Dr. Humboldt, in his testimony before the Sanitary Commission of New Orleans, says:

"I am of opinion that in all countries which can engender fever, the yellow fever is its most concentrated form. I believe it intimately allied in its nature and its causes to ordinary fevers."

According to the inoculator's own showing, it is next to certain that the vipers acted merely a melo-dramatic part; and that not a single particle of the ophidian venom could have remained, as such, in the putrid liver at the time of using the revolting fluid. It is well known that all the animal secretions, when excited to putrefaction, become completely and rapidly decomposed. It is, therefore, pretty clear that the inoculator might just as well have avoided this *coup de theatre*, and plied his patients with his carrion alone, as to have complicated his experiments by mixing it up with the vipers. New Orleans filth—said to be surpassing powerful—would have answered equally well for all that can be believed to the contrary.—*New Orleans Medical and Surgical Journal.*

